# Kinetic Constraints in the Specific Interaction between Phosphorylated Ubiquitin and Proteasomal Shuttle Factors

**DOI:** 10.3390/biom11071008

**Published:** 2021-07-10

**Authors:** Ling-Yun Qin, Zhou Gong, Kan Liu, Xu Dong, Chun Tang

**Affiliations:** 1 Innovation Academy for Precision Measurement Science and Technology, Chinese Academy of Sciences, Wuhan 430071, China; qly_wh@wipm.ac.cn (L.-Y.Q.); gongzhou@wipm.ac.cn (Z.G.); liuk@apm.ac.cn (K.L.); 2 University of Chinese Academy of Sciences, Beijing 100049, China; 3 Beijing National Laboratory for Molecular Sciences, College of Chemistry and Molecular Engineering, Beijing 100871, China; 4 Peking-Tsinghua Center for Life Sciences, Peking University, Beijing 100871, China

**Keywords:** protein-protein interaction, ubiquitin, phosphorylation, proteasomal shuttle factor, ubiquitin-associated domain, induced fit

## Abstract

Ubiquitin (Ub) specifically interacts with the Ub-associating domain (*UBA*) in a proteasomal shuttle factor, while the latter is involved in either proteasomal targeting or self-assembly coacervation. PINK1 *phosphorylates Ub* at S65 and makes Ub alternate between C-terminally relaxed (*pUb_RL_*) and retracted conformations (*pUb_RT_*). Using NMR spectroscopy, we show that *pUb_RL_* but not *pUb_RT_* preferentially interacts with the *UBA* from two proteasomal shuttle factors Ubqln2 and Rad23A. Yet discriminatorily, Ubqln2-*UBA* binds to *pUb* more tightly than Rad23A does and selectively enriches *pUb_RL_* upon complex formation. Further, we determine the solution structure of the complex between Ubqln2-*UBA* and *pUb_RL_* and uncover the thermodynamic basis for the stronger interaction. NMR kinetics analysis at different timescales further suggests an indued-fit binding mechanism for *pUb-UBA* interaction. Notably, at a relatively low saturation level, the dissociation rate of the *UBA-pUb_RL_* complex is comparable with the exchange rate between *pUb_RL_* and *pUb_RT_*. Thus, a kinetic constraint would dictate the interaction between Ub and *UBA*, thus fine-tuning the functional state of the proteasomal shuttle factors.

## 1. Introduction

The ubiquitin-proteasomal system is essential for maintaining proteostasis in the cell. Substrate proteins conjugated with the ubiquitin (Ub) chain in a particular manner can be targeted to the proteasome for degradation. The targeting process is mediated by the interactions between Ub and the intrinsic Ub receptors in the proteasome [1,2,3]. Ub-modified substrate proteins can also be targeted to the proteasome with the assistance of proteasomal shuttle factors. A shuttle factor contains an Ub-like domain (Ubl) at its N-terminus and one or more Ub-associating domains (*UBA*) at its C-terminus. The Ubl interacts with the same receptors in the proteasome, often with higher affinity than Ub [2,4]. The *UBA*, on the other hand, transiently interacts with Ub. Together, the proteasomal shuttle factor bridges the interaction between the substrate protein and the proteasome.

Ubiqulin-2 (Ubqln2) and Rad23A are the two common proteasomal shuttle factors. Ubqln2 is one of the four proteins, Ubqln1–4, in the ubiquilin family. Ubqln2 is prone to self-assembly to form liquid droplets or insoluble aggregates [5,6] and is associated with amyotrophic lateral sclerosis with frontotemporal dementia [7] and Huntington’s disease [8]. On the other hand, the interaction with Ub via the Ubqln2-*UBA* domain can shift the equilibrium towards the diluted phase and dissipate Ubqln2 coacervate [5]. UV excision repair proteins, including Rad23A and Rad23B, can also phase separate in the cell. But unlike Ubqln2, Rad23 self-assembly is facilitated with the addition Ub chain, resulting in the formation of proteasome foci in the cell [9]. The reason for the distinct coacervation behavior for the two proteasomal shuttle factors can be two-fold. First, a Rad23 protein harbors two tandem *UBA* domains, allowing multivalent interactions with Ub instead of just one *UBA* domain in Ubqln2. Second, Ub has a weaker binding for Rad23 than for Ubqln2 [10,11].

Ub not only modifies other proteins, Ub itself can also be modified [12]. Phosphorylation by kinase PINK1 at Ub residue S65 has been studied most intensively [13,14,15], thanks to its connection to the Parkinson’s disease [16,17]. An increase of S65-phosphorylated Ub (*pUb*) level has been observed in neurons and brains of the aging population and neurodegenerative disease patients [18,19]. It has been suggested that Ub phosphorylation by PINK1 can inadvertently impair proteasomal activity and disrupt proteostasis [15]. Indeed, Ub phosphorylation can interfere with the synthesis and hydrolysis of the Ub chain [20,21,22], i.e., the writers and erasers of the Ub signaling cascade. In comparison, how Ub phosphorylation impacts the noncovalent interactions between Ub and other proteins is less clear [23]. A Ub phosphomimetic has been shown to bind to Rad23A much tighter than the wildtype Ub [21] in a quantitative proteomics study, but it is unknown how the wildtype *pUb* behaves.

When phosphorylated by PINK1 at S65, the resulting *pUb* can undergo a large conformational change to adopt a C-terminal retracted conformation (*pUb_RT_*) [20]. The alternative conformation differs from the typical C-terminal relaxed conformation (*pUb_RL_*), mainly in the hydrogen-bond register of the last β-strand (β5). The two conformational states are in slow exchange and about equally populated at the physiological pH [14,24]. Importantly, mutants mimicking the S65 phosphorylation cannot elicit the alternative conformation [24,25]. To understand how the phosphorylation impacts Ub noncovalent interactions with other proteins, we characterized the binding dynamics and kinetics between *pUb* and the two *UBA* domains from Ubqln2 and Rad23A. Our results indicate that Rad23A does not bind to *pUb* more tightly than to Ub. Moreover, we show that Ubqln2-*UBA* selectively interacts with and enriches *pUb_RL_* owing to a kinetic constraint.

## 2. Materials and Methods

### 2.1. Sample Preparation

Human ubiquitin was cloned to a pET11a vector and expressed in BL21 star cells. LB medium and M9-minimum medium were used to prepare unlabeled proteins and isotope-enriched proteins, respectively. Ubiquitin was purified through Sepharose SP and Sephacryl S100 columns (GE Healthcare, New Brunswick, NJ, USA) in tandem. For isotopic labeling, 1 g/L U-^15^N-labeled NH_4_Cl (Isotec, Kürten, Germany) and/or 2 g/L U-^13^C-labeled glucose (Isotec, Milwaukee, WI, USA) were added to the M9-minimum medium as the sole nitrogen and/or carbon source.

PINK1 from body louse (phPINK1) was prepared as previously described [24]. To phosphorylate Ub, PINK1, Ub, MgCl_2_, and ATP were mixed at the molar ratio of 1:10:500:500 in 20 mM Tris HCl buffer, also containing NaCl 150 mM, 1 mM DTT at pH 8.0. The reaction was performed at room temperature for 4 h. The *pUb* product was further purified with the Source Q column (GE Healthcare, New Brunswick, NJ, USA). Successful phosphorylation of Ub by PINK1 was confirmed by ESI mass spectrometry (Bruker Daltonics, Billerica, MA, USA).

The genes encoding the human Ubqln2-*UBA* (residues 578 to 621) and human Rad23A-*UBA2* (residues 315 to 363) were synthesized with optimized codons and sub-cloned into pET11a plasmid (a thioredoxin tag, a hexahistidine tag, and a TEV cleavage site were appended at the N-terminus of *UBA*). All proteins were expressed using BL21 (DE3) strain. LB medium and M9-minimal medium were used to prepare unlabeled and isotope-enriched proteins, respectively. After cell lysis, the protein was purified with a Ni-NTA agarose column (GE Healthcare, New Brunswick, NJ, USA) and a Sephacryl S100 column (GE Healthcare, New Brunswick, NJ, USA). The tags were removed with TEV protease at 4 °C overnight, followed by a second Ni-NTA agarose column (the desired protein was recovered in the flowthrough) and Sephacryl S100 columns column.

### 2.2. NMR Titration Experiments

For the titration of ^15^N-labeled Ub or *pUb* with Ubqln2-*UBA* or Rad23A-*UBA2*, the initial NMR sample was prepared as 100 μM in 20 mM HEPES buffer (containing 150 mM NaCl at pH 7.4). A series of ^1^H-^15^N HSQC spectra were recorded for the ^15^N-labeled *pUb* sample with Ubqln2-*UBA* or Rad23A-*UBA2* at 22.7 μM, 56.7 μM, 113.5 μM, 141.8 μM, 170.2 μM, 226.9 μM, 283.6 μM and 340.4 μM as the final concentration. The ^1^H-^15^N HSQC spectra were recorded using a Bruker 600 MHz NMR spectrometer (Bruker Daltonics, Billerica, MA, USA) at 25 °C (298 K).

The NMR data were processed and analyzed using NMRPipe [26] and CCPNmr Analysis V2.4 [27], respectively. The CSPs were computed with [0.5 × ΔδH^2^ + 0.1 × ΔδN^2^)]^0.5^, in which ΔδH and ΔδN were the chemical shift difference in ^1^H and ^15^N dimensions, respectively.

### 2.3. Determination of K_D_ Value for pUb-UBA Complex

The zero-order equilibrium between *pU_RL_* and *pUb_RT_* can be written as below:

(1) $$pUb_{RL}\overset{R}{↔}\;pUb_{RT}$$

The ratio between *pUb_RT_* and *pUb_RL_* concentrations is a constant at a particular pH [26]:

(2) $$R=\frac{\left[pUb_{RT}\right]}{\left[pUb_{RL}\right]}\;$$

Now we consider the *UBA* interaction with *pUb_RL_*, and the entire equilibrium can be written as follow:

(3) $$pUb_{RT}\;\overset{R}{↔}\;pUb_{RL}+UBA\;\overset{K_{D\;}}{↔}\;Complex$$

The dissociation constant (*K_D_*) between *pUb_RL_* and *UBA* is defined as:

(4) $$K_{D}=\frac{\left[pUb_{RL}^{free}\right]\times \left[UBA^{free}\right]}{\left[Complex\right]}\;$$

The total concentration of *pUb* [*M*] is:

(5) $$M=\left[pUb_{RT}\right]+\left[pUb_{RL}^{free}\right]+\left[Complex\right]$$

The concentration of *UBA* added [*T*] at a given titration point is known and can be substituted in the following equation:

(6) $$M=\left[pUb_{RT}\right]+\frac{\left[pUb_{RT}\right]}{R}+\left[T\right]-\left[UBA^{free}\right]$$

(7) $$pUb_{RL}^{free}=\frac{\left[pUb_{RT}\right]}{R}=\frac{K_{D}\times \left(\left[T\right]-\left[UBA^{free}\right]\right)}{\left[UBA^{free}\right]}$$

Combining Equations (6) and (7), the [*pUb_RT_*] is described as follow:

(8) $$\left[pUb_{RT}\right]=\frac{-\left\{R\times \left(\left[T\right]-M\right)+K_{D}\times R\times \left(R+1\right)\right\}+\sqrt{{\left\{R\times \left(\left[T\right]-M\right)+K_{D}\times R\times \left(R+1\right)\right\}}^{2}+4\times M\times R^{2}\times K_{D}\times \left(1+R\right)}}{2\times \left(R+1\right)}$$

On the other hand, the sum of [*pUb_RL_*] and the concentration of *pUb_RL_* in the complex form is defined in the following equation:

(9) $$\left[P\right]=\left[pUb_{RL}^{free}\right]+\left[Complex\right]=M-\frac{-\left\{R\times \left(\left[T\right]-M\right)+K_{D}\times R\times \left(R+1\right)\right\}+\sqrt{{\left\{R\times \left(\left[T\right]-M\right)+K_{D}\times R\times \left(R+1\right)\right\}}^{2}+4\times M\times R^{2}\times K_{D}\times \left(1+R\right)}}{2\times \left(R+1\right)}$$

For NMR titration, the relationship between the CSP and protein concentration is described as follow:

(10) $$\delta_{obs}=\delta_{min}+n\times \left(\delta_{max}-\delta_{min}\right)\times \frac{\left(\left[T\right]+\frac{\left[P\right]}{n}+K_{D}\right)-\sqrt{{\left(\left[T\right]+\frac{\left[P\right]}{n}+K_{D}\right)}^{2}-\frac{4\times \left[T\right]\times \left[P\right]}{n}}}{2\times \left[P\right]}$$

in which *δ_obs_* is the observed CSP value at a given titration point, *n* is the stoichiometric ratio, [*P*] is the total concentration of *pUb* relaxed state, *δ_min_* is the minimum value of CSP, *δ_max_* is the maximal value of CSP. [*T*] is the concentration of the *UBA* domain. In Equation (10), [*P*] is described using Equation (9), the value of *K_D_* for the complex between *UBA* and *pUb* relaxed state could be calculated by fitting the concentration of *UBA* [*T*] against observed CSP *δ_obs_*.

### 2.4. CPMG Relaxation Dispersion Experiment

^15^N-edited CPMG measurement was performed for the NMR sample of the complex between Ubqln2-*UBA* and *pUb* prepared as concentrations (in 150 mM NaCl, 20 mM HEPES pH 7.4 buffer); one with 32 μM *pUb* and 300 μM Ubqln2 *UBA* (~10% saturation), and the other with 228 μM *pUb* and 300 μM Ubqln2 *UBA* (~50% saturation). The complex samples of Rad23A-*UBA2* and *pUb* were also prepared with 54 μM *pUb* and 300 μM Rad23A-*UBA2* (~5% saturation). The NMR sample of the complex between Ubqln2-*UBA* and ^15^N-*pUb* prepared as concentrations (in 20 mM HEPES pH 7.4 buffer with 150 mM NaCl); one with 39 μM Ubqln2 *UBA* and 300 μM ^15^N-*pUb* (~10% saturation), and the other with 229 μM Ubqln2 *UBA* and 300 μM ^15^N-*pUb* (~50% saturation). The CPMG experiments were recorded using Bruker 600 MHz, 700 MHz, and 850 MHz NMR spectrometers (Bruker Daltonics, Billerica, MA, USA) using the standard pulse sequence [28]. The CPMG spin-echo pulsing frequency includes 0 Hz, 40 Hz, 120 Hz, 200 Hz, 280 Hz, 360 Hz, 600 Hz, and 760 Hz. The NMR data were processed using NMRPipe and fitted using Glove [29].

The concentration of the free *pUb_RL_* can be calculated using Equations (2) and (4). The relationship between *K_D_*, *k_ex_*, *k_on_*, and *k_off_* is described in Equations (11) and (12), and thus can be calculated.

(11) $$k_{ex}=k_{on}\times \left[pUb_{RL}^{free}\right]+k_{off}$$

(12) $$K_{D}=\frac{k_{off}}{k_{on\;}}$$

### 2.5. Acquisition of NMR ZZ-Exchange Data

^15^N-labeled *pUb* (380 μM) and Ubqln2-*UBA* (280 μM) were mixed in 20 mM HEPES buffer at pH 7.4 containing 150 mM NaCl. As a control, 380 µM free ^15^N-labeled *pUb* was prepared in the HEPES buffer. The experiments were performed on a Bruker 600 MHz NMR spectrometer (Bruker Daltonics, Billerica, MA, USA) at 30 °C. The delay times for the ZZ-exchange were set at 0 ms, 20 ms, 40 ms, 60 ms, 90 ms, 120 ms, 160 ms, 220 ms, 380 ms, and 450 ms. The signal intensities with different delays were evaluated and used to fit the exchange rates between *pUb_RL_* and *pUb_RT_*, using the established method [30].

### 2.6. Calculation of the UBA-pUb Complex Structure

The ^13^C-edited F1-filtered NOESY spectra were recorded with a 120 ms mixing time on a 600 MHz NMR spectrometer at 25 °C. The ^15^N/^13^C-labeled Ubqln2-*UBA* (500 μM) and *pUb* (750 μM) were mixed in 20 mM HEPES buffer pH 7.4 with NaCl 150 mM. For the RDC sample, ^15^N-labeled *pUb* (300 μM) and Ubqln2 *UBA* (700 μM) were mixed in the same buffer. Residual dipolar couplings (RDC) were recorded for backbone amide bond vectors in PEG (C_12_E_5_)/hexanol (6%; Sigma-Aldrich, Saint-Louis, MO, USA) alignment medium [31], using the in-phase/anti-phase scheme.

The structure of the complex between the Ubqln2 *UBA* and the *pUb_RL_* was calculated using XPLOR-NIH [32]. The topology and parameter files for phosphorylated serine (SEP) were generated as previously described [14]. For the intra-molecular restraints of Ubqln2 *UBA* (PDB code: 2JY6) and the *pUb* relaxed state (PDB code: 5XK5), the published data were used during the structure determination [24]. Intermolecular NOE and RDC restraints were used to restrain the complex structure. The RDC restraints of *pUb_RL_* with Ubqln2-*UBA* (85% complex at *pUb* and Ubqln2-*UBA* concentrations of 300 µM) and its free form were recorded separately in the same alignment medium. To determine the RDC values of *pUb_RL_*/Ubqln2 *UBA* complex, the contribution of the free form of *pUb_RL_* was subtracted from the observed data of the sample of *pUb_RL_* with Ubqln2 *UBA*, using the following equation:

(13) $$RDC_{complex}=\frac{\left(RDC_{complex-measured}-RDC_{free-measured}\times \%free\right)}{\%complex}$$

Two hundred forty structures each were calculated, and the top-ranked 20 structures with the lowest energy were selected. The structures were further subjected to water refinement. for further analysis. Structure figures were rendered using PyMOL Version 2.2 (The PyMOL Molecular Graphics System, Schrödinger). The complex structure of Ubqln2-*UBA* and *pUb_RL_* has been deposited at the PDB with the accession number of 7F7X.

## 3. Results

### 3.1. UBA Selectively Interacts with pUb_RL_

**Figure 1:**
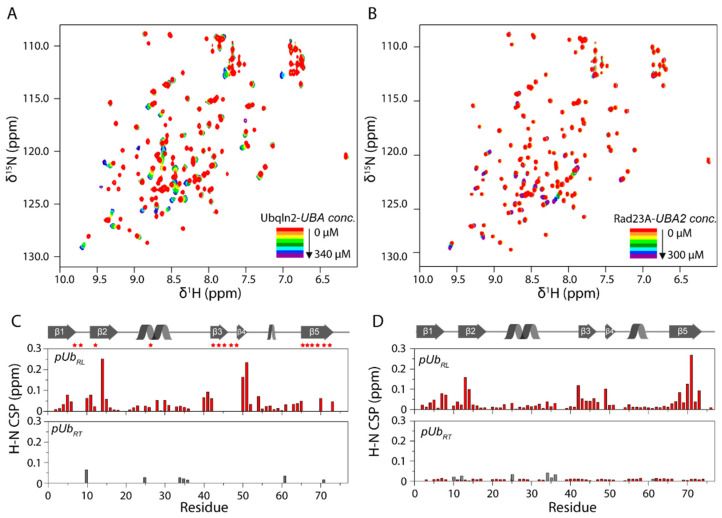
NMR titrations show that Ubqln2-*UBA* and Rad23A-*UBA2* selectively interact with *pUb_RL_*. (**A**,**B**) A series of ^1^H-^15^N HSQC were recorded to monitor the amide chemical shift changes upon titration of Ubqln2-*UBA* and Rad23-*UBA2*, respectively, and the spectra for different titration points are colored like a rainbow. (**C**,**D**) Chemical shift perturbations (CSPs) for the backbone amide of 100 μM ^15^N-labeled *pUb_RL_* and *pUb_RT_* upon titration of 340 μM Ubqln2-*UBA* and 300 μM Rad23A-*UBA2*, with the secondary structure of *pUb* shown as a cartoon. The amide signals that disappear in the complex are indicated with asterisks. The gray columns indicate the residues of *pUb_RL_* and *pUb_RT_* have the same chemical shifts.

We titrated the unlabeled *UBA* domain from Ubqln2 or the second *UBA* domain (*UBA2*) from Rad23A to ^15^N-labeled *pUb*. The titration causes progressive chemical shift perturbations (CSPs) for a subset of peaks in *pUb_RL_* but almost negligible CSPs for the peaks corresponding to *pUb_RT_*. Though the CSP magnitude is similar for *pUb_RL_* when titrated with two *UBA* domains, the largest perturbed residues are found in β4 and β2 with the Ubqln2-*UBA* β5 and with the Rad23A-*UBA2* (Figure 1).

**Figure 2:**
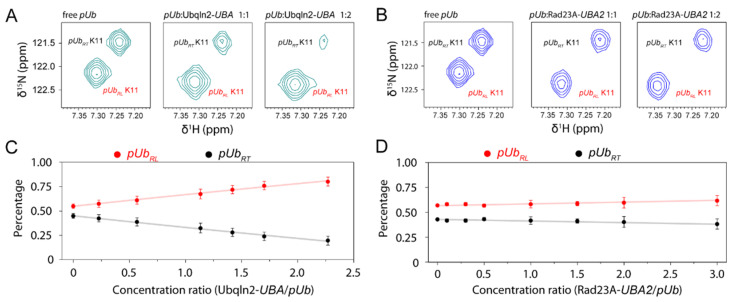
NMR titrations show that Ubqln2-*UBA* selectively enriches *pUb_RL_* at the expense of *pUb_RT_*. The zoomed-in view of ^1^H-^15^N HSQC shows the peaks of K11 of *pUb_RL_* and *pUb_RT_* at the different molar ratio with Ubqnl2-*UBA* (**A**) and Rad23A-*UBA2* (**B**). (**C**,**D**) The populations of *pUb_RL_* (red) and *pUb_RT_* (black) are extracted from the titration of Ubqnl2-*UBA* and Rad23A-*UBA2*. The values are calculated as the percentages of the peak volume of the *pUb* conformational state over the combined peak volume of both *pUb* states, averaged for four residues at the Ub-*UBA* interface; the error bar indicates one standard deviation. The plot shows that the population of *pUb_RL_* increases upon the addition of Ubqln2-*UBA* but barely changes upon the addition of Rad23A-UBA2.

Ubqln2-*UBA* not only selectively interacts with *pUb_RL_* and also enriches *pUb_RL_* to nearly 100%. When binding to Ubqln2-*UBA*, *pUb* undergoes a further equilibrium shift from *pUb_RT_* to *pUb_RL_*. In comparison, the addition of Rad23A-*UBA2* causes little populational change for the *pUb*, even with the addition of a very high concentration (Figure 2).

**Figure 3:**
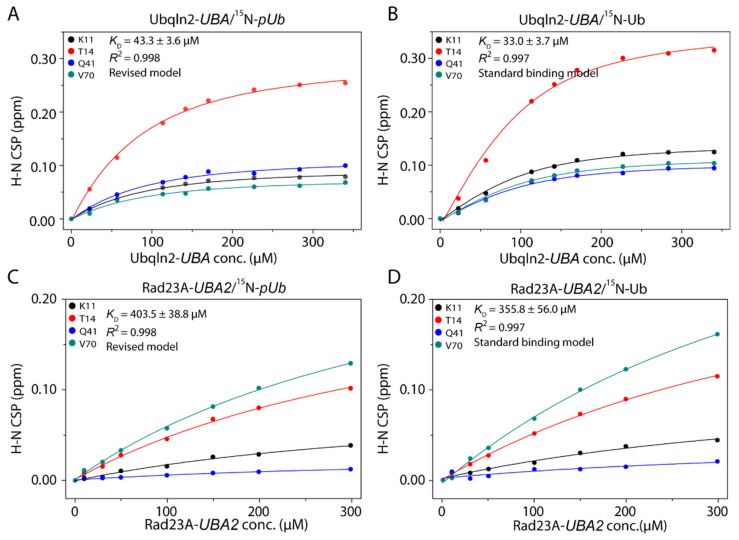
Fitting of the binding equilibrium constants between *UBA* and *pUb_RL_* using our revised model. The interfacial residues were selected for their largest and continuously visible CSPs. (**A**,**C**) Fitting the CSPs of ^15^N-labeled *pUb* upon the titration of Ubqln2-*UBA* and Rad23A-*UBA2*. The *K_D_* values are 43.3 ± 3.6 μM and 403.5 ± 38.8 μM, respectively. (**B**,**D**) Fitting the CSPs of ^15^N-labeled Ub upon the titration of Ubqln2-*UBA* and Rad23A-*UBA2* using a simple one-site binding model. The *K_D_* values are 33.0 ± 3.7 μM and 355.8 ± 56.0 μM, respectively.

In the standard one-site binding isotherm model, the concentration of the titrated protein is fixed. In a previous study by Fushman and coworkers, the titration points between *pUb* and the *UBA* domain from Ubqln1, a close homolog of Ubqln2 in the same family, were fitted with a simple one-site binding curve [25]. However, systematic deviations can be noticed from the fitting (Figure S1). Upon *UBA* titration, the total *pUb_RL_* concentration changes, while the unbound *pUb_RL_* concentration should maintain a constant ratio with the *pUb_RT_* concentration. Thus, a revised model is needed to account for the coupled equilibria of the interconversion between *pUb_RL_* and *pUb_RT_* and the interaction between *pUb_RL_* and *UBA*, as described in the Methods section. Using this model, we obtained the *K_D_* values of 43.3 ± 3.6 µM and 403.5 ± 38.8 µM for Ubqln2-*UBA* and Rad23A-*UBA2*, respectively (Figure 3A,C). Notably, the residuals are small and random from the fittings (Figure S2).

As a control, we performed titrations of Ubqln2-*UBA* and Rad23A-*UBA2* to ^15^N-labeled unmodified Ub. The *K_D_* values are 33.0 ± 3.7 µM and 355.8 ± 56.0 µM for Ubqln2-*UBA* and Rad23A-*UBA2*, respectively. Thus, phosphorylation only slightly decreases the binding affinities of the *UBA* towards the *pUb_RL_* (Figure 3B,D).

### 3.2. The Complex Structure Explains the Binding Preference for Ubqln2-UBA

We collected the intermolecular NOEs between *pUb* and Ubqln2-*UBA* using a filtered/edited NMR pulse sequence. Consistent with the titration results, the NOE cross-peaks were only identified between the resonances associated with *pUb_RL_* and the resonances in Ubqln2-*UBA* (Figure S3, Table S1). On the other hand, we could not observe intermolecular NOEs between *pUb* and Rad23A-*UBA2*. The failure to produce intermolecular NOEs can be explained by the short lifetime, i.e., fast dissociation rate, of the Rad23A-*pUb* complex, as will be discussed below. In addition to the NOEs, experimental restraints also include residual dipolar couplings (RDCs), measured for each subunit at an exact complex occupancy.

**Figure 4:**
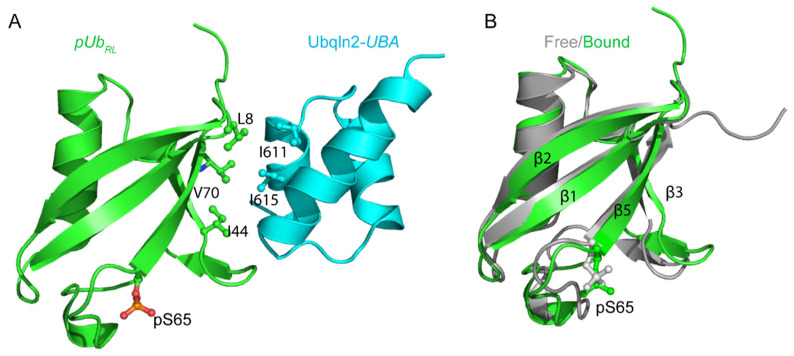
Solution structure of *pUb_RL_* in complex with Ubqln2-*UBA* (PDB code: 7F7X, this study). (**A**) The structure of the complex between Ubqln2-*UBA* (cyan) and *pUb_RL_* (green), with the pS65 sidechain shown as sticks. The key interfacial residues, including L8, I44, and V70 in *pUb_RL_*, and I611 and I615 in Ubqln2-*UBA* are also shown. (**B**) Superposition of *pUb_RL_* structure in the free form (gray) or complex with Ubqln2-*UBA* (green). The pS65 sidechains are shown as sticks.

The structure of the complex between *pUb_RL_* and Ubqln2-*UBA* is well-converged with the root-mean-square deviations for all backbone heavy atoms of 0.81 ± 0.09 Å (Figure S4 and Table S2). The structure is similar to those determined for other *UBA*-Ub complexes [11,33]. Formation of the complex buries solvent-accessible surface area of 1022.7 ± 139.7 Å^2^, which involves hydrophobic residues of L8, I44, and V70 in *pUb_RL_* and hydrophobic residues I611 and I615 in Ubqln2-*UBA* (Figure 4A). Moreover, the β-sheet slightly bends upon complex formation, as the distance between the Cβ atoms of residues I44 and V70 is shortened from 6.3 ± 0.2 Å in the free *pUb_RL_* to 5.8 ± 0.2 Å in the *UBA*-bound *pUb_RL_*. Since β5 moves up by two residues in *pUb_RT_* [20,24], the interaction between V70 in *pUb_RL_* and I615 in Ubqln2-*UBA* would be abolished. This explains why the *UBA* selectively interacts with *pUb_RL_*.

The phosphorylated residue pS65 is located outside the interface between *pUb_RL_* and Ubqln2-*UBA*. This explains why Ubqln2-*UBA* binds to *pUb_RL_* only slightly weaker than the unmodified Ub (Figure 4A). Interestingly, the interface does not involve residues in β2. Therefore, the observed CSP for β2 residues (Figure 1) is likely caused by the allosteric modulation of the β-sheet structure upon Ubqln2-*UBA* binding. Indeed, significant changes in the RDC values are observed for the interfacial residues and the backbone N-H bond vector of β2 residue L15 (Figure S5). As a result, β1 and β2 strands curl slightly in the *UBA* complex (Figure 4B).

Rad23A-*UBA2* is highly homologous to Ubqln2-*UBA*. However, the interfacial residues I611 and I615 in Ubqln2-*UBA* are substituted with glutamate and alanine residues, respectively, in Rad23A-*UBA2*. Therefore, Rad23A-*UBA2* interacts with *pUb_RL_* much weaker than Ubqln2-*UBA*. Moreover, Rad23A-*UBA2* likely adopts a slightly different conformation in the complex, which would explain the different CSP profile (Figure 1).

### 3.3. Kinetic Constraints for pUb Interaction

The addition of Ubqln2-*UBA* enriches *pUb_RL_*. However, the addition of Rad23-*UBA* largely failed to promote *pUb* conformational conversion. To account for the different selectivity for the *UBA* domain, we performed a detailed kinetics analysis during the formation of the *pUb-UBA* complex.

**Figure 5:**
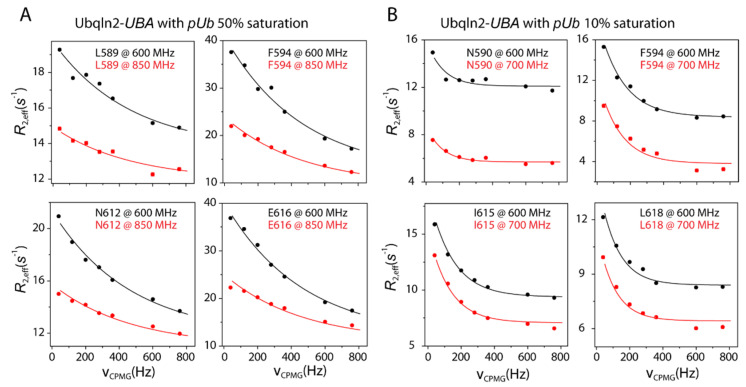
CPMG relaxation dispersion analysis for ^15^N-labeled Ubqln2-*UBA* at two different magnetic fields. The unlabeled *pUb* was added to ~10% and ~50% saturation. Experimental values of *R_2,eff_* at different CPMG frequencies are shown as dots, with the error bar indicates one standard deviation in the measurement.

We first performed CPMG relaxation dispersion measurement for ^15^N-labeled Ubqln2-*UBA*, with the unlabeled *pUb* added to ~10% and ~50% saturation of the complex (Figure 5). The measurements were performed at two different magnetic fields, and the *k_ex_* values for the exchange rate between free and bound proteins can be obtained in a global fit. At 1382.9 ± 3.7 s^−1^ and 320.3 ± 13.9 s^−1^, the *k_ex_* value is nearly four times larger at the higher concentration level. Using the *k_ex_* values and the *K_D_* value (the zero-order interconversion equilibrium constant between *pUb_RL_* and *pUb_RT_* is set to 0.67), we obtained the concentration of the unbound *pUb_RL_* and determined the *k_on_* and *k_off_* values—6.8 ± 0.6 µM^−1^ s^−1^ and 293.1 ± 27.5 s^−1^ at the low saturation level, and 15.6 ± 1.4 µM^−1^ s^−1^ and 677.8 ± 61.0 s^−1^ at the high saturation level.

Reciprocally, we performed CPMG relaxation dispersion measurements for ^15^N-labeled *pUb*, with the unlabeled Ubqln2-*UBA* added at ~10% or ~50% saturation. However, the fitting was poor, especially at 10% saturation. We managed to obtain the *k_ex_* value using residue L71 in the *pUb_RL_* conformational state (Figure S6A,B), and the exchange rate at 50% saturation is also much higher than that at 10% saturation. As a control, we performed CPMG relaxation dispersion measurement for ^15^N-labeled *pUb* alone. The change in the ^15^N *R*_2_ value for L71 in the free *pUb* is negligible, indicating a lack of µs-ms timescale, which has been noted for the unmodified Ub [34].

Binding to Ubqln2-*UBA* also perturbs the exchange dynamics between *pUb_RL_* and *pUb_RT_*. The interconversion between the two Ub states occurs at ms-s timescale and can be probed with ZZ-exchange spectroscopy [20]. Binding to Ubqln2-*UBA* causes slight retardation of the back exchange from *pUb_RL_* to *pUb_RT_* compared to the free *pUb* (Figure S7).

We also performed CPMG relaxation dispersion measurements for the ^15^N-labeled Rad23-*UBA2* with the unlabeled *pUb* added to ~5% saturation. The ^15^N *R*_2_ values only decreases slightly at an increasing CPMG pulsing frequency (Figure S8). Thus, only a lower limit could be estimated for the *k_ex_* value for the exchange rate between free and bound forms, which is ~40,000 s^−1^.

## 4. Discussion

Ub is an important signaling molecule and performs its function by modifying other proteins, the post-transitional modification process known as ubiquitination. Discoveries made in the past ten years have shown that Ub itself can be modified, and Ub modifications can profoundly remodel Ub signaling [12,15]. Ub is phosphorylated at residue S65 by PINK1 [13]. The *pUb* slowly interconverts between two distinct conformational states, namely *pUb_RL_* and *pUb_RT_* [20]. Using NMR titrations, we have shown that the *UBA* from Ubqln2 and Rad23A selectively interacts with *pUb_RL_* but not *pUb_RT_*. We have thus characterized the complex structure between Ubqln2-*UBA* and *pUb_RL_* and provided an atomic explanation for the *UBA* selectivity of the particular *pUb* state. Interestingly, Ubqln2-*UBA* selectively enriches *pUb_RL_* at the expense of *pUb_RT_* during the interaction. Prompted by this finding, we revised the one-site binding model to account for the changing concentration of *pUb_RL_* during *UBA* titration. The *K_D_* values from the fitting show that thermodynamically, the *UBA* binding to *pUb_RL_* is only slightly weaker than to the unmodified Ub. This is consistent with the fact that *pUb_RL_* and Ub are structurally similar, and the phosphorylated residue is away from the binding interface (Figure 4).

The selective enrichment of *pUb_RL_* by Ubqln2-*UBA* not only has to do with the thermodynamics of the binding equilibrium but has to do with the association/dissociation kinetics. The interconversion between the free and bound forms for Ubqln2 complex is much slower than that for the Rad23A complex. A slower off-rate means a longer lifetime for the Ubqln2-*pUb* complex, allowing intermolecular NOEs to build up (Figure S3). The slower interconversion rate also means that Ubqln2-*UBA* is more capable, kinetically, of driving the conversion from *pUb_RT_* to *pUb_RL_*.

However, the experimentally determined *k_on_* and *k_off_* rates of the Ubqln2-*pUb* complex are larger than the interconversion timescale between *pUb_RT_* and *pUb_RL_* by over an order of magnitude. Interestingly, the *k_on_* and *k_off_* rates decrease when the saturation level of the complex is lower. Upon extrapolation, the *k_off_* rate would be even lower at the start of the binding process, comparable to the timescale of *pUb_RT_*/*pUb_RL_* interconversion. Moreover, upon Ubqln2 binding, the back conversion from *pUb_RL_* to *pUb_RT_* is slightly slowed (Figure S7). Together, owing to the kinetic constraint, *pUb_RL_* can be efficiently enriched by Ubqln2 but not by Rad23A.

The acceleration of the binding kinetics at higher complex occupancy can be explained by the conformational restriction of *pUb_RL_*. Ub undergoes a so-called pincer-like movement, with the residues in β1 and β2 experience large fluctuation at sub-µs timescale [35,36]. The association of a *UBA* stabilizes one of the preexisting conformations of the β-sheet structure. However, Ub does not simply bind to *UBA* through conformational selection; an induced fit or conformational restriction mechanism also plays an important role in the interaction between Ub and its partner protein, especially towards the end of the binding process [37,38]. The increased exchange rate between free and bound proteins at an increasing saturation level of the complex is characteristic of an induced-fit mechanism [39]. Microscopically, the interaction with Ubqln2-*UBA* induces and stabilizes a *UBA*-complementary conformation of *pUb_RL_* (Figure 4), which would permit rapid association and dissociation. In a sense, Ub phosphorylation by PINK1 can be likened to Ub mutations specifically introduced that ultimately drive the Ub-binding mechanism to induced fit [40]. Nevertheless, a precise dissection of the two binding mechanisms warrants further analysis [41,42].

As such, phosphorylation at Ub residue S65 provides additional kinetic and dynamic constraints for Ub-*UBA* noncovalent interactions, which would determine whether the proteasomal shuttle factor remains monomeric for proteasomal targeting or phase-separate to form liquid or solid coacervate.

## Data Availability

The data have been deposited with the PDB and BMRB with the accession codes of 7F7X and 36427, respectively.

## Supplementary Materials

The following are available online at https://www.mdpi.com/article/10.3390/biom11071008/s1, Figure S1: Reanalysis of the NMR titration data between *pUb* and the *UBA* domain of ubiquilin1, published by Fushman and coworkers [25]; Figure S2: (A,C) The residuals from the fitting the CSPs of ^15^N-labeled *pUb* upon *UBA* titration, using the revised model, related to Figure 3A,C. (B,D) The residuals from the fitting the CSPs of ^15^N-labeled Ub upon *UBA* titration, using the simple one-site binding model, related to Figure 3B,D; Figure S3: Intermolecular NOEs identified between ^13^C,^15^N-labeled Ubqln2-*UBA* and unlabeled *pUb_RL_*; Figure S4: Superposition of the 20 lowest-energy conformers calculated for Ubqln2-*UBA* (cyan) and *pUb_RL_* complex (green), shown in two perspectives; Figure S5: Comparison between observed and calculated RDC values for *pUb_RL_*; Figure S6: CPMG relaxation dispersion measurements were performed for ^15^N-labeled *pUb* at two different magnetic fields; Figure S7: The interconversion rate between *pUb_RL_* and *pUb_RT_* analyzed with NMR ZZ-exchange experiment; Figure S8: CPMG relaxation dispersion measurements performed for ^15^N-labeled Rad23A-*UBA2* at 600 MHz with the unlabeled *pUb* added to ~5% saturation; Table S1: Intermolecular distance restraints for the structure calculation of Ubqln2-*UBA*/*pUb_RL_* complex.; Table S2: Structure statistics for the Ubqln2-*UBA*/*pUb_RL_* complex; Table S3: The fitted values of *k_ex_*, *k_on_*, and *k_off_* of *pUb_RL_*/Ubqln2 *UBA* complex.
