# Supplementary material for: Kinetic Constraints in the Specific Interaction between Phosphorylated Ubiquitin and Proteasomal Shuttle Factors
Source: Biomolecules. 2021 Jul 10;11(7):1008. doi: 10.3390/biom11071008 (PMC8301994; doi:10.3390/biom11071008)
Supplement: Supplementary file 1 [file biomolecules-11-01008-s001.zip › biomolecules-1252286-supplementary.pdf]

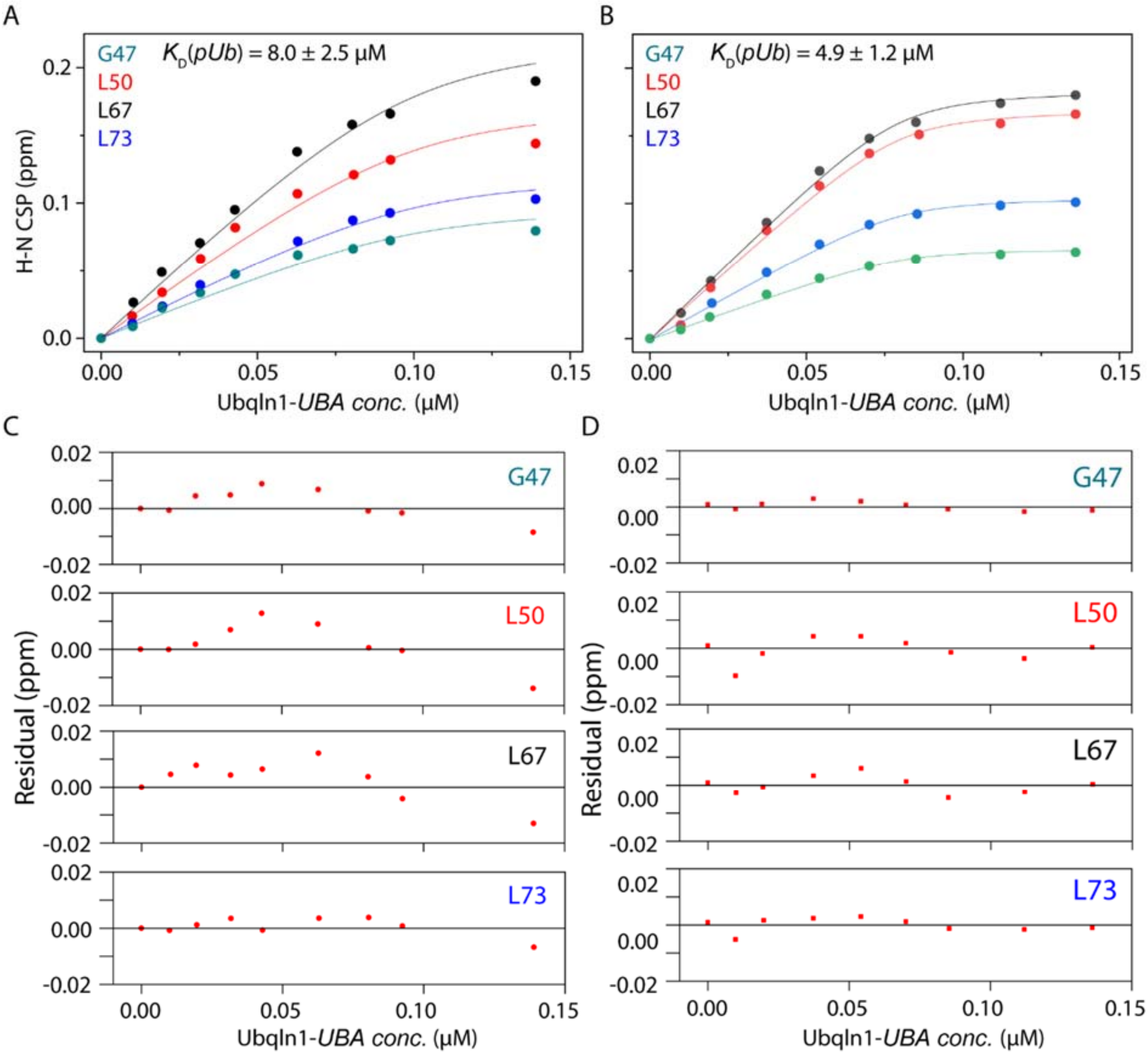

Figure S1. Reanalysis of the NMR titration data between *pUb* and the *UBA* domain of ubiquilin1, published by Fushman and coworkers (*Sci. Rept.* 2018; 8: 2651). The data points were digitized from Figure 3F and 3D of the paper, respectively. The data points were fitted with a simple one-site binding model, and the resulting K_D values are identical, as previously reported. The residuals of the K_D fitting are shown in Panels A and B.

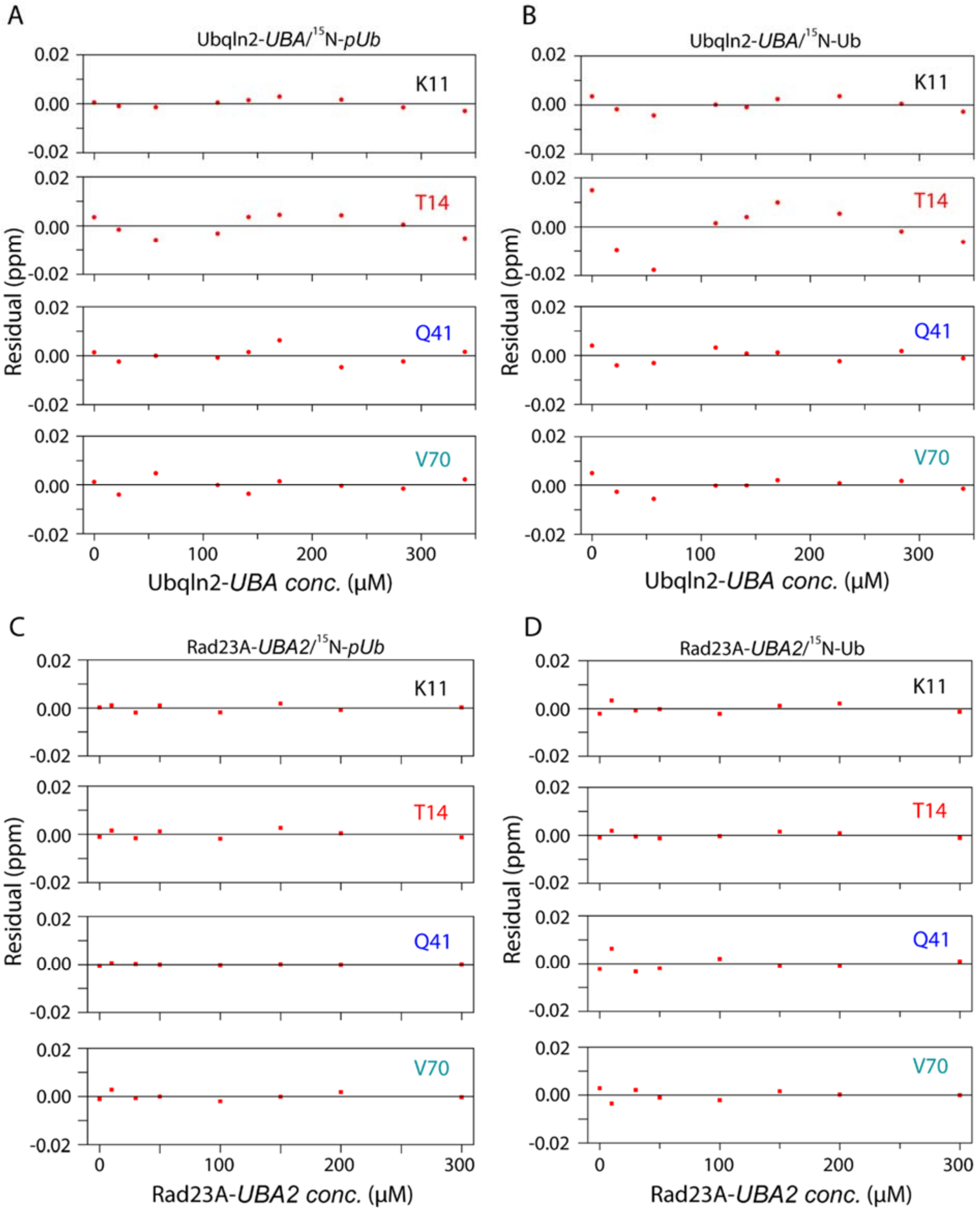

Figure S2. (A, C) The residuals from the fitting the CSPs of ^{15}N-labeled *pUb* upon *UBA* titration, using the revised model, related to Figure 3A, C. (B, D) The residuals from the fitting the CSPs of ^{15}N-labeled Ub upon *UBA* titration, using the simple one-site binding model, related to Figure 3B, D.

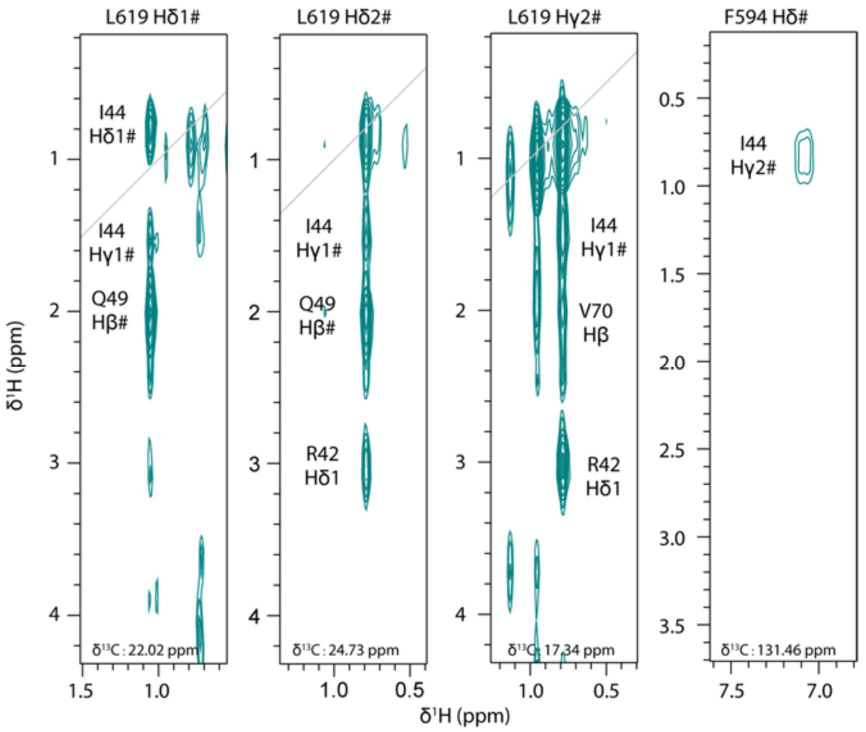

Figure S3 Intermolecular NOEs identified between ^{13}C,^{15}N-labeled Ubqln2-*UBA* and unlabeled *pUb*$_{RL}$. The assignments are labeled here in the F1 (^{1}H) and F2 (^{1}H) dimensions for the intermolecular NOEs in the F3 (^{13}C)-filtered NOESY.

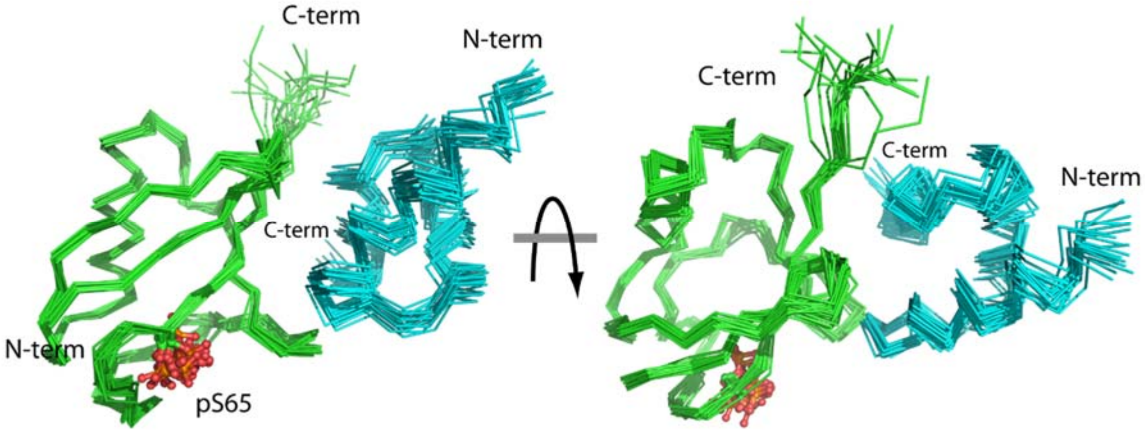

Figure S4 Superposition of the 20 lowest-energy conformers calculated for Ubqln2-*UBA* (cyan) and pUb_{RL} complex (green), shown in two perspectives. The structures are superimposed by backbone heavy atoms of pUb_{RL}. The RMS deviation is 0.61 ± 0.10 Å for the backbone heavy atoms. The phosphorylated residue pS65 is shown as sticks.

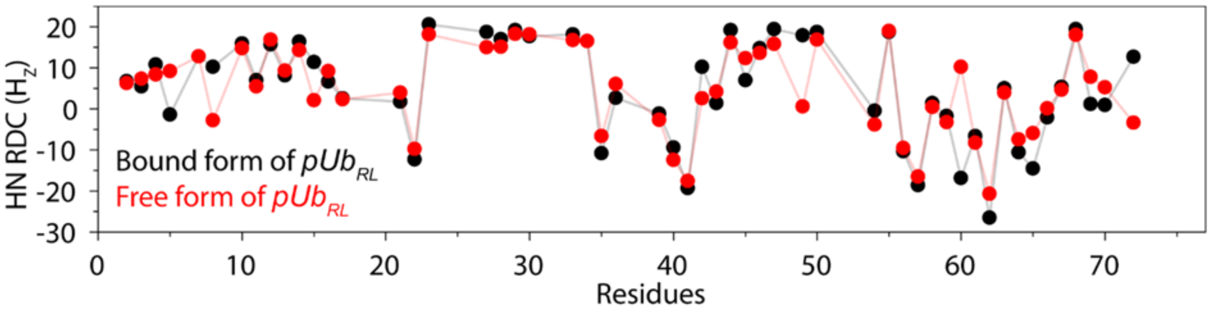

Figure S5. Comparison between observed and calculated RDC values for *pUb*$_{RL}$. The RDC values were measured for backbone amide bond vectors of *pUb*$_{RL}$ in the free form and the complex with Ubqln2-*UBA* in the PEG-hexanol alignment medium extrapolated to 100% of the complex. The free *pUb*$_{RL}$ structure (PDB code 5XK5) was used to calculate the theoretical RDCs using singular value decomposition.

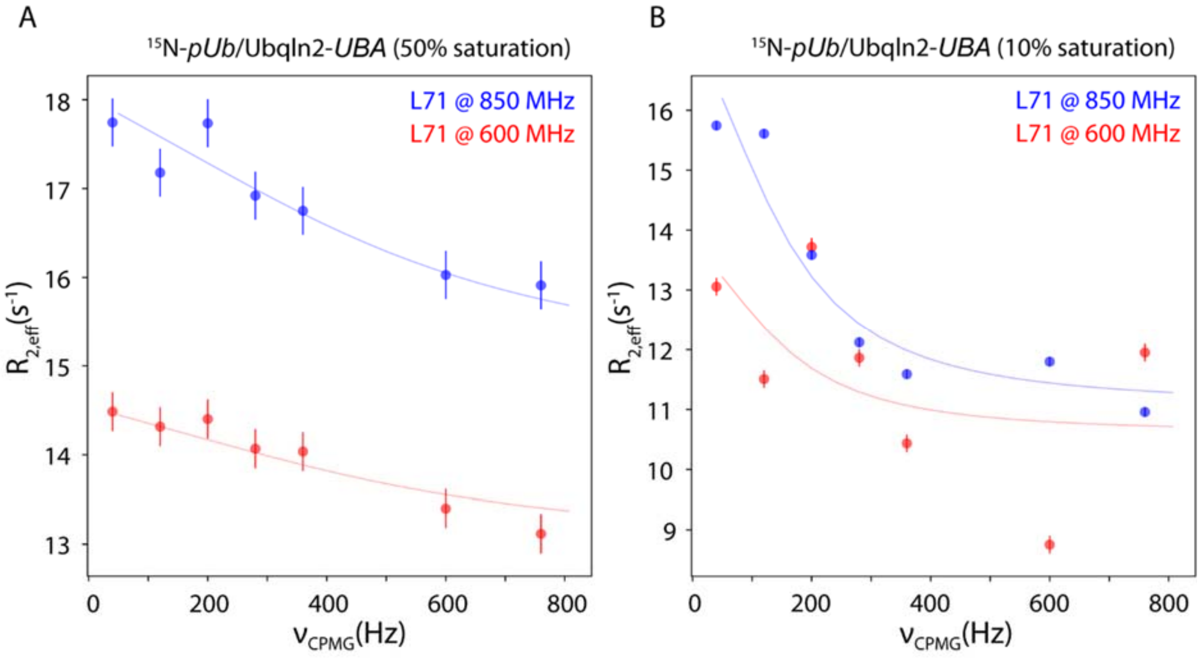

Figure S6. CPMG relaxation dispersion measurements were performed for ^{15}N-labeled *pUb* at two different magnetic fields. The unlabeled Ubqln2-*UBA* was added to ~10% and ~50% saturation. Experimental values of $R_{2,\mathrm{eff}}$ at different CPMG frequencies are shown as dots, with the error bar indicates one standard deviation in the measurement.

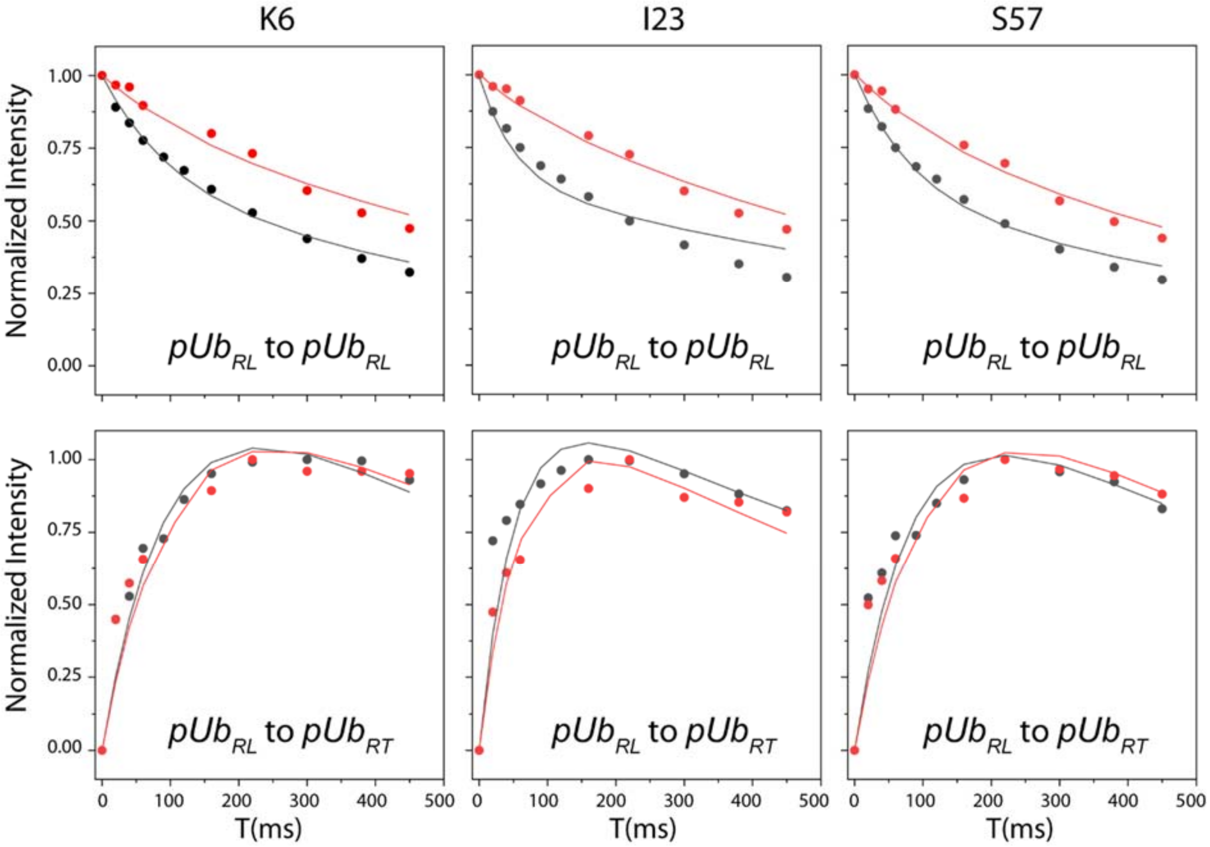

Figure S7 The interconversion rate between pUb_{RL} and pUb_{RT} analyzed with NMR ZZ-exchange experiment. The measurements were performed for either free *pUb* or *pUb* associated with Ubqln2-*UBA* (380 µM and 280 µM, respectively, at 30 ºC). Ubqln2-*UBA* binding causes small perturbation to the interconversion rate; the interconversion rate from pUb_{RL} to pUb_{RT} is 16.96 s^{-1} in free pUb and 14.92 s^{-1} in the *pUb* complex.

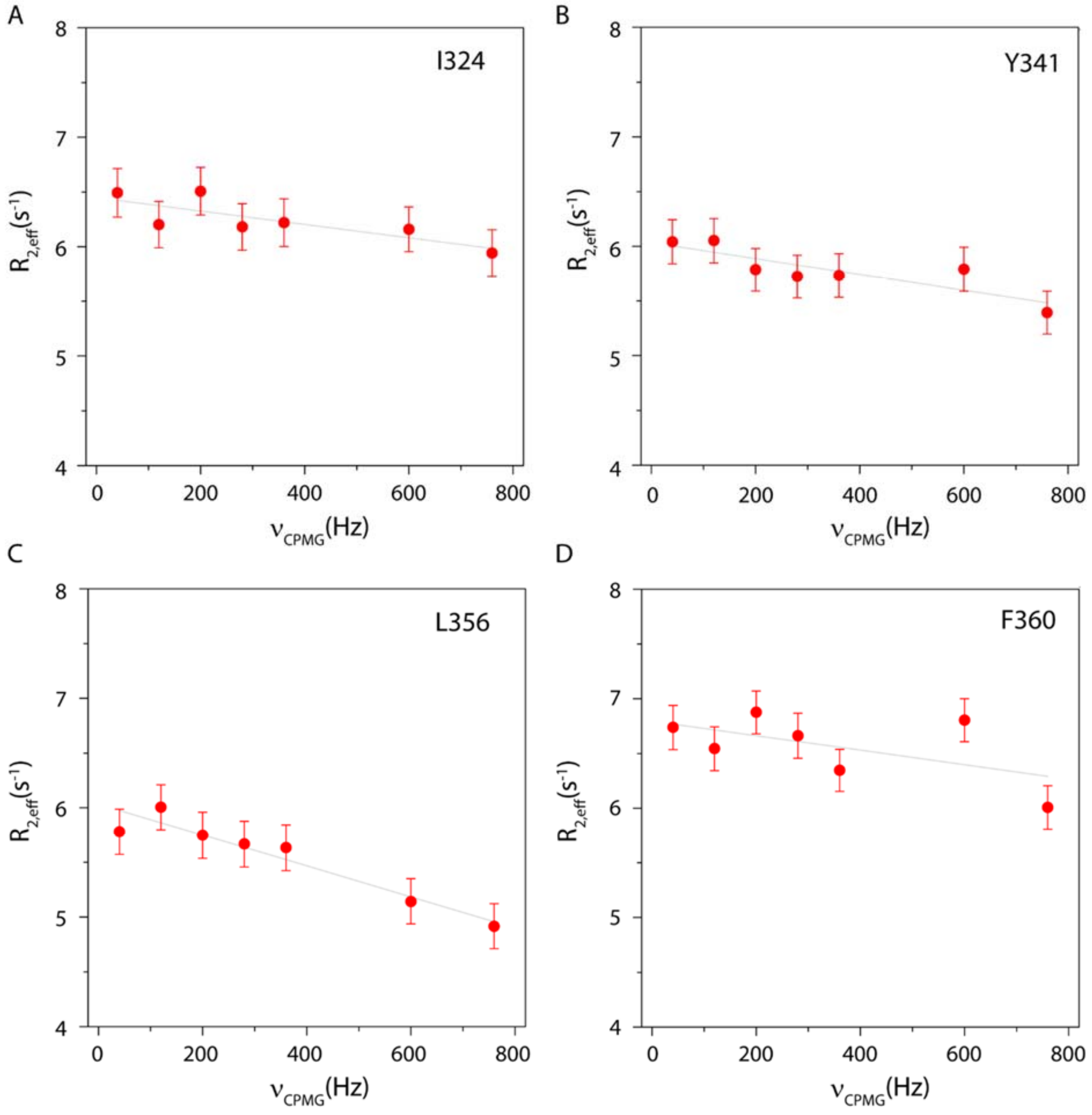

Figure S8 CPMG relaxation dispersion measurements performed for ^{15}N-labeled Rad23A-*UBA2* at 600 MHz with the unlabeled *pUb* added to ~5% saturation. Experimental values of $R_{2,eff}$ at different CPMG frequencies are shown as dots, with the error bar indicates one standard deviation in the measurement.

Table S1 Intermolecular distance restraints for the structure calculation of Ubqln2-*UBA*/pUb_{RL} complex.

Residue 1	Atom 1	Residue 2	Atom 2	Distance/Å
593	HA1	44	HG2#	4.3
593	HA2	44	HG2#	4.1
594	HD#	44	HG2#	3.9
594	HE#	70	HG2#	3.6
612	HB1	70	HG2#	3.3
612	HB1	72	HA	4.2
612	HB1	72	HG2	3.8
612	HB2	72	HA	4.2
612	HB2	72	HG2	3.8
612	HB2	8	HD2#	3.3
615	HG2#	44	HG12	3
615	HG2#	68	HB#	3.5
615	HG2#	70	HB	3.2
615	HG2#	70	HG1#	3
619	HD1#	42	HA	4.2
619	HD1#	44	HD1#	2.9
619	HD1#	44	HG1#	3.4
619	HD1#	49	HB#	2.7
619	HD2#	42	HD1	3.2
619	HD2#	44	HG1#	3.3
619	HD2#	49	HG2	2.9

Table S2 Structure statistics for the Ubqln2-*UBA*/pUb_{RL} complex.

**Number of restraints**	
NOE	1867
Intra-molecular (*pUb* relaxed state)	330
Intra-molecular (Ubqln2-*UBA*)	1521
Inter-molecular	16
RDC	
PEG HN (*pUb* relaxed state)	55
PEG HN (*Ubqln2-UBA*)	35
PEG HNCO (*pUb* relaxed state)	34
H-bond	52
Dihedral angles	
φ/ψ	100
**Structure statistics**	
Number of violations (per structure)	
Distance constraints (> 0.5 Å)	0
Dihedral angle constraints (> 5º)	0
RDC correlation	
HN (*pUb* relaxed state)	0.999±0.000
HN (*Ubqln2-UBA*)	0.996±0.001
HNCO (*pUb* relaxed state)	0.953±0.010
**Average Pairwise r.m.s deviation (Å)**	
Back-bone atoms	0.81± 0.09
All atoms	1.20± 0.07
**Ramachandran plot (aa:1-72)**	
most favored	85.5%
additionally allowed	11.8%
generously allowed	0.8%
disallowed	0.9%
**Z-scores (aa:1-72)**	
2nd generation packing quality	-1.102
Ramachandran plot appearance	-2.929
χ^1/χ^2 rotamer normality	-2.315
Backbone conformation	-0.646

Table S3 The fitted values of k_{ex}, k_{on}, and k_{off} of *pUb*$_{RL}$/Ubqln2-*UBA* complex. These data were calculated by fitting corresponding CPMG data of ^{15}N-Ubqln2-*UBA*/*pUb* and ^{15}N-*pUb*/Ubqln2-*UBA* samples when ~10% and ~50% saturated.

Sample	Complex %	k_{ex} (s^{-1})	k_{on} ($\mu M^{-1}\cdot s^{-1}$)	k_{off} (s^{-1})
^{15}N-labeled *UBA*	10%	320.3±13.9	6.8±0.6	293.1±27.5
	50%	1382.9±3.7	15.6±1.4	677.8±61.0
^{15}N-labeled *pUb*	10%	506.2±4.0	9.8±0.9	426.1±38.5
	50%	1694.2±9.4	14.3±1.3	618.2±55.6

The parameter and topology files for the phosphorylated Ser (SEP) are shown as follows:

########################## Topology ######################################

```
 residue SEP
  group
    atom N   type=NH1 charge=-0.36 end
    atom HN  type=H   charge= 0.26 end
 group
    atom CA  type=CT  charge= 0.00 end
    atom HA  type=HA  charge= 0.10 end
 group
    atom CB  type=CT  charge= 0.08 end
    atom HB1 type=HA  charge= 0.10 end
    atom HB2 type=HA  charge= 0.10 end
 group
    atom OG  type=OH  charge=-0.68 end
 group
    atom P   type=XP  charge= 1.88 end
    atom O1P type=XO2 charge=-0.40 end
    atom O2P type=XO2 charge=-0.40 end
    atom O3P type=XO2 charge=-0.40 end
 group
    atom C   type=C   charge= 0.48 end
    atom O   type=O   charge=-0.48 end

  bond N  HN
  bond N  CA     bond CA HA
  bond CA CB     bond CB HB1     bond CB HB2
  bond CB OG     bond OG P
  bond P O1P     bond P  O2P     bond P O3P
  bond O  C
  bond C  CA

  improper HA N C CB
  improper HB1 HB2 CA OG

  dihedral OG  CB  CA  N
  dihedral  P  OG  CB  CA
```

######################## Parameter ######################################

```
 bond      XP   OH              $kbon     1.537
 bond      XP   XO2             $kbon     1.567
 angle    OH   XP   XO2         $kang    106.7
 angle    CT   OH   XP          $kang    120
 angle    XO2  XP   XO2         $kang    115
 dihedral  XP   OH   CT   CT      $kdih    3    0.0
 NONBonded  XP      0.5849   3.3854      0.5849   3.3854
 NONBonded  XO2     0.2304   2.7290      0.2304   2.7290
```
